# Antifouling coating based on biopolymers (PCL/ PLA) and bioactive extract from the sea cucumber *Stichopus herrmanni*

**DOI:** 10.1186/s13568-022-01364-3

**Published:** 2022-02-27

**Authors:** Mehrnoosh Darya, Mehdi Haji Abdolrasouli, Morteza Yousefzadi, Mir Masoud Sajjadi, Iman Sourinejad, Maaroof Zarei

**Affiliations:** 1 Department of Fisheries, Faculty of Natural Resources, University of Guilan, Sowmeh Sara, Iran; 2 Department of Chemical Engineering, Faculty of Chemical Engineering, University of Hormozagn, Bandar-Abbas, Iran; 3 Department of Biology, Faculty of Science, University of Qom, Qom, Iran; 4 Department of Fisheries, Faculty of Marine Science and Technology, University of Hormozgan, Bandar Abbas, Iran; 5 Department Of Chemistry, Faculty of Basic Science, University Of Hormozgan, Bandar Abbas, Iran

**Keywords:** Antibacterial, Biofouling, Biodegradable, Cytotoxicity, Sea cucumber

## Abstract

An important challenge to decrease the toxic effects of the common biocides in marine environments and to achieve suitable ecofriendly natural antifouling coatings is to find effective natural antifoulants and efficient biodegradable coatings. In this study, antifouling activities of nine bioactive extracts (non-polar to polar) from different organs of the sea cucumber *Stichopus herrmanni* were tested against five bacterial strains, barnacle and brine shrimp larvae. The ethyl acetate extract of the body wall showed the highest in-vitro antifouling activity including high antibacterial and anti-barnacle activities and low toxicity against the brine shrimp as non-target organism. Based on these results, 10 phr of the ethyl acetate extract from *S.herrmanni* was added to different coatings consisting of polycaprolactone (PCL)/polylactic acid (PLA) blends containing various compositions of PLA (0, 10, and 20 wt.%). Polyvinyl chloride panels were coated with the prepared antifouling coatings and immersed in seawater for three months. Panel coated with PCL 80% /PLA 20% containing 10 phr of the antifoulant (panel-5), showed the highest resistance against fouling settlement with fouling coverage of 41.66% (*P* < 0.05). In addition, the lowest fouling weight was measured in panel-5 as well (81.00 ± 9.85 g) (*P* < 0.05). These findings indicate the antibacterial and antifouling potential of semi-polar bioactive extracts from the *S. herrmanni* body wall as natural antifoulants, as well as the enhanced antifouling performance of PCL/the natural antifoulant coatings by adding PLA.

**Graphical Abstract:**

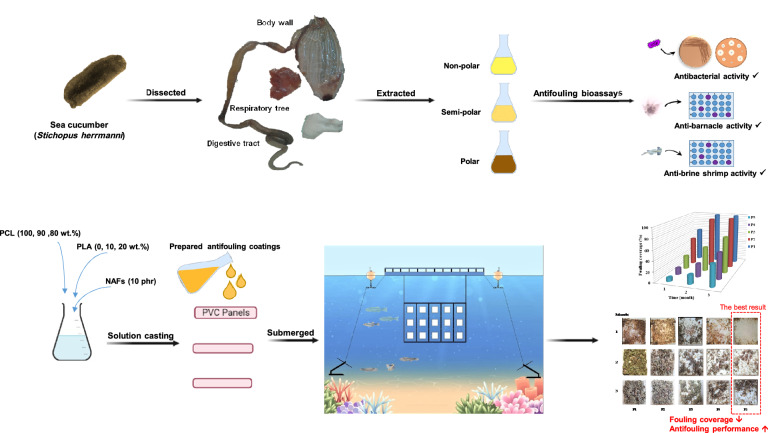

## Introduction

Marine biofouling is a worldwide issue causing an important environmental impact and serious economic and health problems to aquaculture industries and other marine structures (Suresh et al. 2016; Silva et al. 2021). Biofouling directly affects both the aquaculture target species and infrastructure (Fitridge et al. 2012). So far, many strategies have been used in order to control the effects of biofouling, but only a few of them have been practical enough to be widely used for a long time; although they were not harmless. For many years, tributyltin (TBT) and other organotin compounds were the most widely used active agents in antifouling coatings and paints. However, they are harmful to the marine environment because they can leach into the ecosystem and cause harm to non-target organisms (Gittens et al. 2013; Iyapparaj et al. 2013). Accordingly, taking into account the environmental damages and health problems of TBT, International Maritime Organization (IMO) banned its use in September 2008 (Qian et al. 2010).

Following the ban of TBT, metal biocides like zinc and copper have been presented as alternatives to TBT. Copper has been the most used biocide in antifouling coats in recent years (Jerabek et al. 2016), generally with Cu_2_O as the active agent in a polymer matrix (Gittens et al. 2013). Nevertheless, due to serious negative impacts of toxic metal biocides on marine environment and aquaculture species (including bioaccumulation) (Yamada 2007; Bao et al. 2008), there is an urgent need to find nontoxic, environment-friendly and effective antifouling compounds. To discover such proper antifoulants, marine organisms (mostly invertebrates and algae) that typically remain free of foulers have been widely investigated (Qian et al. 2015). These organisms possess secondary metabolites as a chemical defense against biofouling (da Gama et al. 2008; Piazza et al. 2011; Acevedo et al. 2013).

Sea cucumbers are a group of marine benthic invertebrates that produce natural products to defend themselves against external threats. Natural products from Sea cucumbers have been widely investigated for their biological activities such as antibacterial (Mashjoor and Yousefzadi 2017), antialgal, antifungal (Han et al. 2009; Mashjoor and Yousefzadi 2017), antitumoral (Tian et al. 2005) and so forth. Terpenoids are one of the most investigated secondary metabolites of the sea cucumbers, which may be used as new possible antifouling agents (Mert Ozupek and Cavas 2017; Darya et al. 2020). In this regard, triterpene glycosides and saponins derived from the sea cucumbers have been previously considered as suitable candidates for cytotoxic, antimicrobial and antifouling agents (Aminin et al. 2015; Mert Ozupek and Cavas 2017; Bahrami et al. 2018).

Antifouling agents can be incorporated into the polymer matrix to form a composite coating (Palanichamy and Subramanian 2017). This coating can allow the antifoulant to move gradually from the surface leading to prevent deposition and settlement of the fouler (Azemar et al. 2015). Biobased and/or biodegradable polyesters such as Polycaprolactone (PCL) have the potential to be used as a carrier in marine antifouling composite coatings because they can be decomposed and hydrolyzed by microorganisms or enzymes in the seawater, which results in self-renewing surface (Faÿ et al. 2006). However, the drawback of low rate of degradation can interfere the antifouling ability (Xu et al. 2014; Xie et al. 2019). Some studies have focused on the combination effects between the degradation occurred in composite coatings which can be enhanced through physical and/or chemical modification and allowing the antifoulant to move from the surface. (Yao et al. 2014) developed an antifouling coating based on PCL/nanoclay containing an organic antifoulant (4,5-Dichloro-2-octyl-isothiazolone (DCOIT)). Marine field test showed that the antifouling properties and durability of the surfaces were improved by coating with PCL/nanoclay/DCOIT. Biodegradable PCL based polyurethane coating containing butenolide (which is derived from marine bacteria) was introduced by Ma et al. (2017). They found that the system had a great antifouling ability lasting for more than 3 months. Chen et al. (2016) prepared PCL/ poly(butylene succinate) (PBS) blends containing 10.0 wt% of organic antifoulant (4,5-dichloro-2-octyl-isothiazolone) by solution casting for anti-fouling coating. They showed that incorporation of PBS in PCL increased the degradation and releasing rate of the organic antifoulant. It is also interesting to note that polylactic acid (PLA) is a biocompastable and renewable bioplastic which can be used in eco-friendly antifouling strategies (Thouvenin et al. 2003). Azemar et al. (2020) synthesized the block copolymers containing PLA with high antifouling efficiency. Kamtsikakis et al. (2017) used a PLA for encapsulation of three different organic biocides for antifouling application. Chiang et al. (2020) developed a poly(lactic acid)-based polyurethane as a carrier of antifouling coating with optimized release of antifoulant.

In this study, we tried to deal with the biofouling problems in the marine environment in order to decrease the harmful and toxic effects of common biocides. Four different combinations of biodegradable biopolymers were used as coatings and carriers for natural antifoulants. Therefore, we developed biodegradable coatings based on Polycaprolactone (PCL)/Polylactic acid (PLA) containing bioactive compounds extracted from the sea cucumber *Stichopus herrmanni*, and evaluated their antifouling properties by designing marine field tests.

## Materials and methods

### Sea cucumber collection and extracts preparation

Specimens of the sea cucumber *S.herrmanni* were collected by scuba diving from the coastal waters of Hengam island, the Persian Gulf, Iran. After anaesthetizing with magnesium chloride (5%), the sea cucumbers were washed with fresh water, dissected and their body parts and organs (including body wall, digestive tract and respiratory tree) were separated (Mamelona et al. 2007). Each part was freeze-dried and grinded by an electric grain mill. Afterwards, a serial extraction was done based on the solvent polarity using *n*-hexane (non-polar), ethyl acetate (semi polar) and methanol (polar). About 200 g of each part was extracted serially based on increasing solvent polarity at 20–23 ℃ for 48 h in darkness. The obtained extracts were filtered through Whatman paper No. 42 for the removal of residual solids and concentrated under reduced pressure to remove the solvents.

### Biodegradable polymer materials

Polycoaprolactone (PCL), Capa 6800 with a melt flow rate of 3 g/10 (190 ^0^C at 2.16 kg), was purchased from Perstorp. Polylactic acid (PLA), 2003D with a melt flow rate of 5–7 g/10 (210 ^0^C at 2.16 kg) was supplied by Natureworks LLC.

### Antibacterial assay

Antibacterial activity of the sea cucumber extracts were tested against five Gram-positive/negative bacterial strains: *Staphylococcus aureus* (ATCC 25923), *Micrococcus luteus* (ATCC 9341), *Escherichia coli* (ATCC 25922), *Klebsiella pneumoniae* (ATCC 10031) and *Vibrio harveyi* (ATCC 14126); which were obtained from the Pasteur Institute, Tehran, Iran.

Antibacterial screening was done by disc diffusion method (Vanden Berghe and Vlietinck 1991). For stock solutions, 10 mg of each extract of *S.herrmanni* were dissolved in 1 mL of DMSO. Mueller–Hinton Agar plates were inoculated with an overnight culture (12–16 h) of each bacterial strain. Whatman paper disks were placed on the agar surface and injected with 10 μL of each stock solution extract. Disks injected with DMSO were used as solvent control. Disks with standard antibacterial agent ampicillin (at 10 µg/disc) were used as positive control. After the 24 h-long incubation at 37 °C, The diameter of the inhibition zone (IZ) of bacterial growth was measured. No inhibitory effects due to the solvent were observed. The assays were repeated in triplicate.

### Anti-barnacle assay

Barnacles (*Amphibalanus amphitrite*), sessile marine arthropods, were collected from intertidal zone in Bandar Abbas, Iran. To obtain barnacle larvae, the adults were air-drained for 12–14 h and then immersed in filtered seawater (salinity 35% and temperature 25 ℃) (Jun et al. 2008). Nauplius larvae released by adults were transferred to a glass beaker containing filtered seawater until used for the tests. The nine types of stock extracts were dissolved in DMSO and six concentrations were prepared using a serial dilution method. Then, 200 µl of each dilution and 1800 µl of filleted seawater were pipetted into each well of 24-well plates and 10–20 barnacle larvae were added. The final concentrations of each extract in the wells were: 1, 0.5, 0.25, 0.125, 0.062 and 0.031 mg/mL. The 24-well plates were incubated in darkness at 26 ± 1 ℃ (Venkatnarayanan et al. 2016). After 24 h, the number of larvae that had died or were still alive was counted. Median lethal concentration (LC_50_) values were calculated based on Probit analysis. As a control, the same assay was done without extract solution (extract dissolved in DMSO). In addition, DMSO was used instead of extract solution as a negative control. No inhibitory effects due to the solvent were observed. All concentrations (including controls) were tested in triplicate.

### Brine shrimp cytotoxicity assay (as non-target organism)

Brine shrimp (*Artemia salina*) cysts were purchased and hatched in cone-shaped tanks filled with filtered seawater. Artemia nauplii were hatched at 28 ± 2 °C with continues illumination and aeration (Maruthanayagam et al. 2013). Cytotoxicity assay against *A. salina* was also done with the same concentration and control as the barnacle cytotoxicity assay. No inhibitory effects due to the solvent were observed. Similar to the previous experiment, the LC_50_ was calculated for each extract against the artemia larvae.

### Preparation of PCL/PLA/natural antifoulant coating

The film coatings varying in component ratio were prepared by solution mixing. First, PCL and PLA were dissolved by stirring in chloroform (CHCl_3_) at 50 °C. The weight ratio of PCL to PLA were chosen as 100:0, 90:10 and 80:20. Then 10 phr (part per hundred resin/polymer) of the ethyl acetate extracted-compounds of the body wall from sea cucumber *S. herrmanni*, as natural antifoulant, were added into PCL/PLA solutions while stirred for 2 h. At last, the solutions were coated on polyvinyl chloride (PVC) panels (10 × 10 cm) and left to dry at room temperature for 10 days to remove the solvent.

### Thermal properties

Differential scanning calorimetry was performed with a NETZSCH DSC Polyma 214 to study nonisothermal crystallization of the samples at a heating/cooling rate of 10 ℃/min between 0 ℃ and 180 ℃. The samples were first heated from 0 ℃ to 180 °C and held for 5 min. Then, they were cooled from 180  to 0 ℃. The basic equation for calculating the percentage of crystallinity (Xc) of the hybrid materials via DSC is as follows:

$$ X_{c} = \frac{{\Delta H_{c} }}{{\varphi \Delta H_{100} }} $$

where $\Delta H$ is the crystallization enthalpy of the samples; $\varphi$ is the weight percent of PCL; $\Delta H_{100}$ is enthalpy of crystallization for a 100% crystalline PCL, which is taken as 136.0 J/g in this study (Rezgui et al. 2005).

### Marine field antifouling assay

Panels were set in handmade frames and hung on a cage (marine cage culture system) at a depth of 2–3 m in north of the Persian Gulf in Bandar Gorzeh, Hormozgan, Iran for three months. Frames (including coated and uncoated panels) were immersed vertically at the depth of 2 m from the water surface and fixed using buoy and anchor. The prepared panels were included panel-1 (P1): uncoated, panel-2 (P2): coated with PCL (100%), panel-3 (P3): coated with PCL 100% / antifoulant 10 phr, panel-4 (P4): coated with PCL 90% /PLA 10% / antifoulant 10 phr and panel-5 (P5): coated with PCL 80% /PLA 20% / antifoulant 10 phr. The antifouling performance of polymer coats was evaluated monthly by photograph recording (Chen et al. 2016; Soliman et al. 2017) and weighing of settled fouling on panels. The coverage percentage of fouling that settled on panels was calculated using image software (CPCe).

### Statistical analysis

Statistical differences in diameter of IZs and weight of the settled foulings were determined by one-way analysis of variance (ANOVA), using Duncan’s multiple range test. Difference significances were evaluated at *P* < 0.05. The LC_50_ values were calculated by probit analysis using EPA probit analysis program. The fouling cover percentage was estimated by dot-grid method and CPCe 4.1 program.

## Results

### Antifouling laboratory bioassays

#### Antibacterial activity

The antibacterial activity results of *S. herrmanni* bioactive extracts, evaluated by disk diffusion method, are presented in Table 1. Baron and Finegold (1990) described a method for measurement of antimicrobial activity. Based on this method, among the nine bioactive extracts, *n*-hexane and ethyl acetate extracts from the body wall showed high antibacterial activity against Gram-positive bacterium *S. aureus* with inhibition zone higher than 14 mm.

**Table 1:** Antibacterial activity of sea cucumber *S. herrmanni* extracts by disk diffusion method (inhibition zone, mm)

Microorganism	*n*-hexane extracts			Ethyl acetate extracts			Methanol extracts			Ampicillin^a^
	Body wall	Digestive tract	Respiratory tree	Body wall	Digestive tract	Respiratory tree	Body wall	Digestive tract	Respiratory tree	
*S. aureus*	14.70 ± 0.26^b^	10.77 ± 0.33^e^	7.97 ± 0.21^h^	15.27 ± 0.26^a^	11.87 ± 0.29^d^	10.07 ± 0.17^f^	11.97 ± 0.21^d^	8.60 ± 0.33^g^	7.46 ± 0.21^i^	14.17 ± 0.13^c^
*M. luteus*	8.93 ± 0.21^c^	7.33 ± 0.13 ^f^	_	11 ± 0.25^b^	8.47 ± 0.05^d^	7.33 ± 0.13^f^	9.03 ± 0.13^c^	7.97 ± 0.21^e^	7.43 ± 0.17^f^	12.07 ± 0.13 ^a^
*V. harveyi*	9.37 ± 0.30 ^c^	_	_	11.30 ± 0.25^a^	8.63 ± 0.26^d^	7.10 ± 0.08^f^	10.03 ± 0.21^b^	7.43 ± 0.25^e^	_	11.37 ± 0.09^a^
*E. coli*	8.30 ± 0.27^c^	_	_	10.50 ± 0.22^b^	_	_	8.23 ± 0.25^c^	_	_	12.23 ± 0.20^a^
*K. pneumoniae*	7.13 ± 0.11^c^	_	_	8.63 ± 0.27^b^	_	_	_	_	_	10.00 ± 0.33^a^

Data are shown as the mean ± SD with different letters in the same row are significantly different at *p* < 0.05

Inactive (–), weak active (< 7), moderately active (7–14); highly active (> 14)

Inhibition zone includes diameter of the disc (6.4 mm)

^a^Tested at 10 µg/disc

The ethyl acetate extract of the body wall exhibited significantly higher inhibition zone against *S. aureus* compared with other extracts and ampicillin (control) (*P* < 0.05). It also exhibited significantly higher inhibition zone against *V. harveyi* in comparison with other extracts (*P* < 0.05).

Among the nine *S. herrmanni* extracts, only two extracts (*n*-hexane and ethyl acetate extracts from the body wall) showed inhibition activity (moderate) against *K. pneumoniae*. Therefore, *K. pneumoniae* was the most resistant tested bacteria.

Overall, only the ethyl acetate and *n*-hexane extracts of the body wall showed growth inhibition activity against all tested bacterial strains.

#### Anti-barnacle activity

As shown in Table 2, both ethyl acetate and methanol extracts of the body wall showed moderate toxicity against *A. amphitrite* larvae, with LC_50_ values of 0.061 & 0.073 mg/mL, respectively. The most pronounced cytotoxic activity against *A. amphitrite* was found in ethyl acetate extract of the body wall, which had the LC_50_ of 0.061 mg/mL. Among the nine extracts tested, *n*-hexane extract of the respiratory tree showed the lowest cytotoxic activity against barnacle larvae, with LC_50_ of 0.361 mg/mL.

**Table 2:** The toxicity effects of extracts from sea cucumber *S. herrmanni* against barnacle *A. amphitrite* larvae

Extract	*n*-hexane			Ethyl acetate			Methanol		
	Body wall	Digestive tract	Respiratory tree	Body wall	Digestive tract	Respiratory tree	Body wall	Digestive tract	Respiratory tree
LC_50_ (mg/mL)	0.211	0.296	0.361	0.061	0.117	0.148	0.073	0.179	0.246

### Brine shrimp cytotoxicity

As represented in Table 3, all the nine bioactive extracts showed LC_50_ values more than 0.1 mg/mL against brine shrimp *A. salina*. This means that the toxicity of the extracts against brine shrimp (as non-target organism) is low. Also Liu et al. (2018) concluded that the isolated compounds from *Nerium oleander* with LC_50_ above 0.1 mg/mL had very low toxicity against *A. salina*. Since brine shrimp was considered as a non-target organism for any antifouling approaches and agents, having low toxicity against brine shrimp is preferable for an environmental friendly antifouling agent. If anti-brine shrimp and anti-barnacle results are compared, the tested extracts had higher LC_50_ values against the brine shrimp.

**Table 3:** Toxicity effects of extracts from sea cucumber *S. hermmanni* against brine shrimp *A. salina* larvae

Extract	*n*-hexane			Ethyl acetate			Methanol		
	Body wall	Digestive tract	Respiratory tree	Body wall	Digestive tract	Respiratory tree	Body wall	Digestive tract	Respiratory tree
LC_50_ (mg/mL)	0.425	0.591	0.644	0.136	0.326	0.398	0.163	0.375	0.520

### Determination of crystallinity

**Fig. 1:**
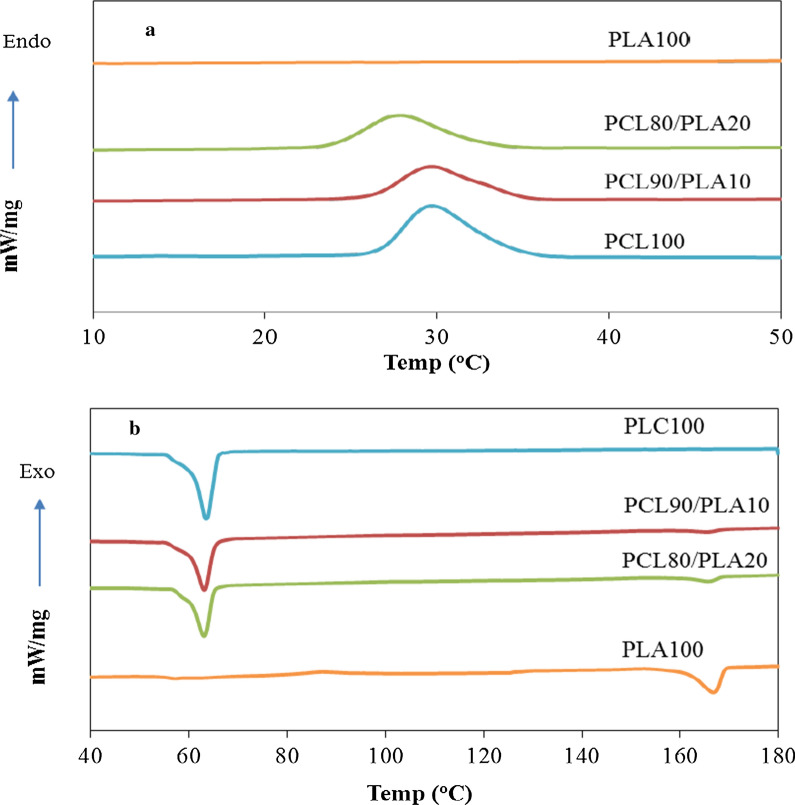
DSC cooling (**a**) and heating (**b**) thermograms recorded for the PCL, PLA and PCL/PLA samples at a rate of 10 ℃/min

DSC analysis was used to determine the crystallinity and melting behavior of the PCL in the PCL/PLA blends at changing compositions. Figure 1 shows the DSC results of virgin PCL, PLA and PCL/PLA samples. Furthermore, the calorimetric parameters of the samples resulted from DSC thermograms are shown in Table 4. The DSC spectra of virgin PCL in cooling (Fig. 1a) exhibits an exothermic crystallization peak at around 30 °C while virgin PLA has shown no peak. It can be seen that crystallization temperature (Tc) and degree of crystalintiy (Xc) of PCL tended to be decreased with the increase of PLA content in PCL/PLA blends. This can be attributed to the formation of defect in PCL crystalline structure and/or increase in amorphous content with the addition of PLA. The crystallization process in polymer blends can be controlled by two competing mechanisms. First, the interfacial surfaces delivered by each phase can lead to increase in the nucleation rate of macromolecules; second, the interfacial interactions between the surfaces of each phase can hinder the motions of molecules leading to the decrease of the growing rate (Luyt and Gasmi 2016). It seems that latter mechanism is dominant in crystallization process of PCL in PCL/PLA blends. In addition, the melting point (T_m_) of PCL crystallite and its enthalpy of melting (${\Delta H}_{m}$**)** obtained from the DSC heating thermogram (Fig. 1 (b)) were also shown in Table 4. The results implied that T_m_ of PCL crystallite and its ${\Delta H}_{m}$ decreased with the addition of PLA.

**Table 4:** DSC Results for PCL, PLA and their blends

Sample	${\Delta H}_{c}$(J/g)	${\Delta H}_{m}$(J/g)	T_c_(^o^C)	T_m_(^o^C)	${X}_{c}$
PCL	58.75	− 70.21	30	64	43.2
PLA	–	− 34.8	–	167	–
PCL 90%/PLA 10%	43.09*	− 49.72*	29.5*	63*	39.60*
PCL 80%/PLA 20%	40.25*	− 43.05*	28*	63*	42.28*

^*^These values are for PCL of the blends

### Antifouling performance (field tests)

The ethyl acetate extract of the body wall showed the highest in-vitro antifouling activity (including antibacterial and anti-barnacle activity); however, due to the limited ecological significance of laboratory bioassays, antifouling activity of a proper candidate should be confirmed by well-designed marine field tests (Soliman et al. 2014). Based on the laboratory results, the ethyl acetate extract of the body wall was mixed with different PCL/PLA biodegradable polymers solution and then were casted on dry PVC panels. The panels coated with polymer films were set on a frame and submerged in marine environment over three months.

**Fig. 2:**
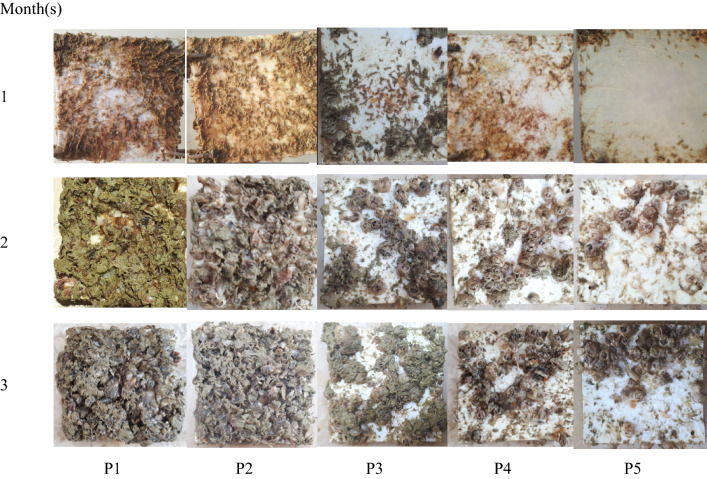
Panels after immersion in the seawater for three months. P1: uncoated panel (control); P2: panel coated with PCL (100%); P3: panel coated with PCL 100% / antifoulant 10 phr; P4: panel coated with PCL 90% /PLA 10% / antifoulant 10 phr; P5: panel coated with PCL 80% /PLA 20% / antifoulant 10 phr

**Fig. 3:**
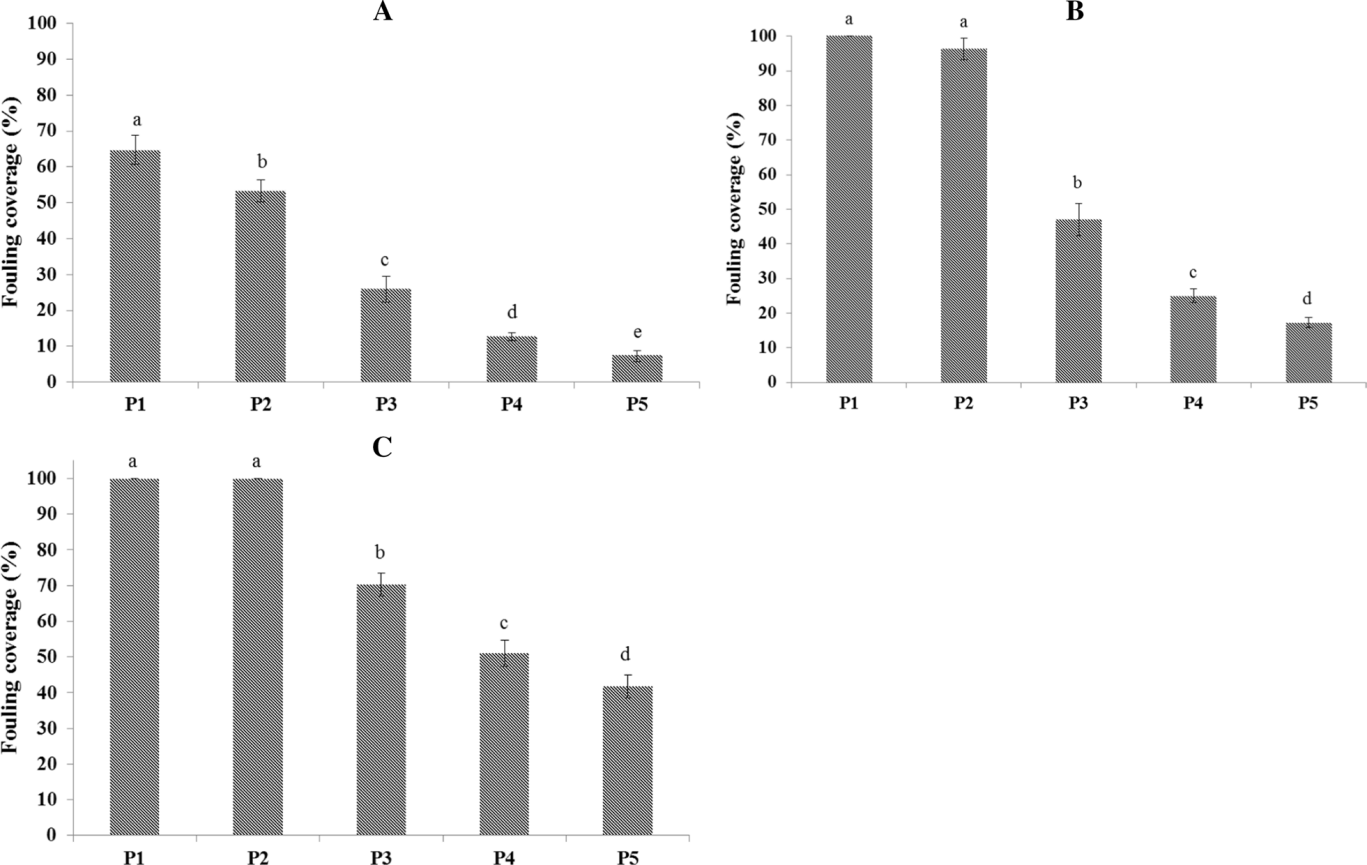
Fouling coverage (%) after immmersion in seawater for one (**A**), two (**B**) and three (**C**) month(s) in Bandar-e-Gorzeh, Persian Gulf. P1: uncoated panel (control); P2: panel coated with PCL (100%); P3: panel coated with PCL 100% / antifoulant 10 phr; P4: panel coated with PCL 90% /PLA 10% / antifoulant 10 phr; P5: panel coated with PCL 80% /PLA 20% / antifoulant 10 phr. Bars with different letters indicate a significant difference (*P* < 0.05) according to Duncan’s multiple range tests

Images of coated and uncoated panels over three months immersion in seawater and also their antifouling performances are shown in Figs. 2 and 3, respectively. A significant increase in fouling coverage (%) on uncoated panel (control) was observed compared with coated panels, especially panel 5, after one month (*P* < 0.05). Fouling coverage showed no significant difference between uncoated and panel coated with PCL (P2), at the end of month two and three (*P* > 0.05). Panel 5 (coated with PCL 80% /PLA 20%/natural antifoulant 10 phr) showed the maximum antifouling performance and the lowest fouling coverage among all the coated panels (*P* < 0.05).

Weight of fouling settled on panels is shown in Table 5. At the end of the experiment, uncoated panel (control) and PCL panel (P2) showed no significant differences in fouling weight (*P* > 0.05). Although, weight of fouling settled on uncoated panel was found to be greatly higher than the coated panels containing bioactive extract of sea cucumber *S. hermmanni* (panel 3, 4 & 5) (*P* < 0.05). Among the coted panels, the panel 5 showed the lowest fouling weight (*P* < 0.05).

**Table 5:** Weight of fouling load on coated panels after immersion in the seawater for three months

PVC panels					
	Uncoated (P1)	P2	P3	P4	P5
Initial panel weight (before immersion) (g)	18.33 ± 1.52	19.33 ± 2.08	20.00 ± 1.73	19.67 ± 2.31	19.67 ± 2.08
Final panel weight (after immersion) (g)	272.67 ± 11.59^a^	259.33 ± 13.80^a^	172.00 ± 13.53^b^	128.00 ± 10.44^c^	100.67 ± 10.12^d^
Fouling weight (g)	254.33 ± 12.50^a^	240.00 ± 12.17^a^	152.00 ± 13.53^b^	108.33 ± 9.29^c^	81.00 ± 9.85^d^

Data are shown as the mean ± SD with different letters in the same row are significantly different at *p* < 0.05

## Discussion

Due to increasing concerns about toxic antifouling biocides, research is now focusing on finding and using natural eco-friendly antifoulants. Natural products from marine invertebrates like the sea cucumbers are found to be promising antifouling agents (Acevedo et al. 2013; Mert Ozupek and Cavas 2017). On the other hand, aliphatic polyesters like polycaprolactone can be used not only as a self-polishing coating but also as a release system for antifoulants due to its enzymatic or hydrolytic erosion (Gross and Kalra 2002; Nair and Laurencin 2007; Patil et al. 2014; Chen et al. 2016; Guo et al. 2016). The present study focused on the use of bioactive extracts from the sea cucumber *S.herrmanni* as natural antifoulants, as well as in the use of the biopolymers PCL/PLA as a self-polishing coating to make an efficient biodegradable antifouling coating.

Since the formation of bacterial biofilms on surfaces is one of the primary stages of biofouling accumulation (Gittens et al. 2013; Xie et al. 2019), antibacterial ability of a natural antifoulant candidate to prevent this accumulation is very important. In the present study, bioactive extracts from the sea cucumber showed a range of low to high antibacterial activities against the tested bacterial strains. Among the nine bioactive extracts of *S.herrmanni,* the ethyl acetate extract of the body wall showed the highest antibacterial activity against all the five bacterial strains. This extract also showed the highest inhibition zone (15.27 ± 0.26 mm) against *S. aureus*, which was significantly higher than the standard antibiotic (ampicillin) (*P* < 0.05). These results are in accordance with a previous study carried out by Mashjoor and Yousefzadi (2017) who showed strong antibacterial activity of several extracts from three different sea cucumbers. Whereas, they reported that the *Staphylococcus epidermidis* was the most sensitive strain against the methanolic and ethyl acetate extracts. In addition, Darya et al. (2020) reported antibacterial and antifouling activates of several extracts (especially ethyl acetate extract) from *Holothuria leucospilota*. They suggested that fatty acids and terpenes as the major detected compounds in the extract might be responsible for antibacterial and antifouling activities.

Barnacles are one of the most important and common macrofouling organisms (Fitridge et al. 2012; Petersen et al. 2020) which highly tend to settle down on the surfaces. The ability to prevent the settlement of barnacles on surfaces can be considered as a crucial factor for a desirable antifouling candidate. Based on antifouling mechanism of action, this prevention can occur through toxic or non-toxic pathways by killing or inhibition of barnacles settlement (Rittschof et al. 1992; Qian et al. 2010). In a study on anti-barnacle activity of alkyl isocyanides derived from citronellol, a natural acyclic monoterpenoid (Kitano et al. 2011), compounds with LC_50_ ranging from 0.021 to 0.07 mg/mL were considered as compounds with moderate anti-barnacle activity. In the present study, ethyl acetate and methanol extracts of the body wall showed moderate anti-barnacle activity with LC50 values of 0.061 and 0.073 mg/mL, respectively. In addition, our results showed that the toxicity of all the nine bioactive extracts were low against brine shrimp, with LC_50_ value of much higher than 0.1 mg/mL. Also, Liu et al. (2018) concluded that the isolated compounds from *Nerium oleander* with LC_50_ above 0.1 mg/mL had very low toxicity against the brine shrimp. Since brine shrimp *A. salina* is considered as non-target organism for any antifouling approaches and agents, having low toxicity against brine shrimp is preferable for an environmental friendly antifouling agent.

All the results of in-vitro antifouling bioassays showed promising antifouling activities for several tested bioactive extracts, especially ethyl acetate extract of the body wall of *S. hermmanni*. Ethyl acetate is a semi-polar organic solvent and widely used to extract bioactive compounds (Asmah et al. 2020). Several studies have indicated that bioactive extracts and compounds from marine invertebrates have good antibacterial, antifungal, cell toxicity and antifouling activities (Tincu and Taylor 2004; Puentes et al. 2014; Gomes Filho et al. 2014; Datta et al. 2015; Ghadiri et al. 2018). Since polarity of the solvent plays a great role in extraction of bioactive compounds (Darya et al. 2020), and because of the economical preference of extraction compared with the purification of compounds (Liu et al. 2018), the selection of a proper solvent is very important to achieve target compounds with antifouling activity and lower price for commercial use. Consequently, ethyl acetate extracted compounds from the body wall were chosen as natural antifoulant for preparing biodegradable antifouling coatings. In line with the present study, Farjami et al. (2013), Mashjoor and Yousefzadi (2017) and Darya et al. (2020) reported antibacterial and antifouling activates from the sea cucumber semi-polar bioactive extracts. In the present study, higher antibacterial and antifouling activities were observed from the ethyl acetate extract of the body wall rather than the viscera. This could be referring to organ-specific distribution of the compounds that are responsible for these biological activities. Bahrami et al. (2018) reported that the distribution of saponins were different between the body wall and viscera of *Holothuria lesson*; and some highly glycosylated saponins were found only in the body wall. They reported that the examined saponins had high antifungal and antioxidant activities.

Due to the high antifouling activity of the ethyl acetate extract of the body wall, it was mixed into PCL/PLA biopolymers solution. Then, the prepared antifouling coatings were casted on PVC panels and submerged in the marine environment for three months. The results of the marine fields showed a very good antifouling performance for ethyl acetate extract as natural antifoulant added to PCL and PCL/PLA coats. Beside, adding 10% & 20% of PLA to the PCL coats could significantly increase antifouling performance of the experimental coats. Among the coated panels, panel coated with PCL 80% /PLA 20% / natural antifoulant from *S. hermmanni* 10 phr had the lowest fouling weight and coverage (settled fouling) after three months. The polymers containing variety of active agents like antifoulants can be used as matrix and/or carrier in coating systems (Azemar et al. 2015, 2020; Xie et al. 2020). In antifoulling coating systems, the erosion and decomposition occurred in the polymer matrix which has degradation properties (like PCL and PLA), resulted in a self-polishing, leading to prevent the biofouling (Pan et al. 2020; Azemar et al. 2020). Furthermore, the higher rate in the controlled release of antifoulant from the polymer matrix of antifouling coatings during the time is desired. The degradable polymers based on PCL and/or PLA matrix which decompose continuously in environments like the seawater can provide the situation in which the antifoulant is released in a controlled manner at higher rate, compared with the undegradable polymers (Yao et al. 2014; Chiang et al. 2020). It seems that the hydrolytic degradation rate in the PCL/PLA coating samples increased with increasing the PLA content as a result of higher hydrolytic degradation rate of PLA than that of PCL due to its lower hydrophobicity (Kutikov and Song 2015). This leads to the higher self-polishing properties and higher releasing rate of antifoulant in the prepared coating samples containing higher content of PLA. On the other hand, permeation of materials such as water, antifoulant and/or impurities through amorphous phase of polymers is easier than that of crystal phase as a consequence of lower compactness (Chen et al. 2016). In addition, it can be expected that the degradation process which facilitates the release of materials can be enhanced with increasing content of amorphous phase in the samples. DSC results showed that the amorphous phase of PCL increased with the addition of PLA and therefore it can more intensify the release of antifoulant from PCL/PLA coating samples. These can be considered as the causes of the highest antifouling properties of PCL 80% /PLA 20% coating containing 10 phr of natural antifoulant from *S. hermmanni.*

Our findings suggest that some bioactive extracts, especially semi polar solvent-extractable compounds, from the sea cucumber *S.herrmanni* might have promising antibacterial and antifouling activities and can be used for environmentally friendly antifouling proposes. We also observed that the antifouling and anti-settling performance of the biopolymer (PCL)/ the natural antifoulant coat could be improved by the addition of PLA. This can be attributed to the enhanced self-renewing properties in combination with the easier releasing of the natural antifoulant.

## Data Availability

The datasets generated during and/or analysed during the current study are available from the corresponding author on reasonable request.
